# IL-33/ST2 Correlates with Severity of Haemorrhagic Fever with Renal Syndrome and Regulates the Inflammatory Response in Hantaan Virus-Infected Endothelial Cells

**DOI:** 10.1371/journal.pntd.0003514

**Published:** 2015-02-06

**Authors:** Yusi Zhang, Chunmei Zhang, Ran Zhuang, Ying Ma, Yun Zhang, Jing Yi, Angang Yang, Boquan Jin

**Affiliations:** Department of Immunology, The Fourth Military Medical University, Xi’an, China; Baylor College of Medicine, UNITED STATES

## Abstract

**Background:**

Hantaan virus (HTNV) causes a severe lethal haemorrhagic fever with renal syndrome (HFRS) in humans. Despite a limited understanding of the pathogenesis of HFRS, the importance of the abundant production of pro-inflammatory cytokines has been widely recognized. Interleukin 33 (IL-33) has been demonstrated to play an important role in physiological and pathological immune responses. After binding to its receptor ST2L, IL-33 stimulates the Th2-type immune response and promotes cytokine production. Depending on the disease model, IL-33 either protects against infection or exacerbates inflammatory disease, but it is unknown how the IL-33/ST2 axis regulates the immune response during HTNV infection.

**Methodology/Principal Findings:**

Blood samples were collected from 23 hospitalized patients and 28 healthy controls. The levels of IL-33 and soluble ST2 (sST2) in plasma were quantified by ELISA, and the relationship between IL-33, sST2 and the disease severity was analyzed. The role of IL-33/sST2 axis in the production of pro-inflammatory cytokines was studied on HTNV-infected endothelial cells. The results showed that the plasma IL-33 and sST2 were significantly higher in patients than in healthy controls. Spearman analysis showed that elevated IL-33 and sST2 levels were positively correlated with white blood cell count and viral load, while negatively correlated with platelet count. Furthermore, we found that IL-33 enhanced the production of pro-inflammatory cytokines in HTNV-infected endothelial cells through NF-κB pathway and that this process was inhibited by the recombinant sST2.

**Conclusion/Significance:**

Our results indicate that the IL-33 acts as an initiator of the “cytokine storm” during HTNV infection, while sST2 can inhibit this process. Our findings could provide a promising immunotherapeutic target for the disease control.

## Author Summary

Hantaan virus (HTNV) causes human hemorrhagic fever with renal syndrome (HFRS) with a mortality rate of approximately 15% in Asia. At present, the primary treatment for HFRS is limited to critical care management and the use of anti-viral drugs, such as Ribavirin. However, the cytokine storm at the acute phase of HFRS, which is thought to contribute to the development of the disease, is still lacking an effective way to prevent. An alternative way to prevent the development of cytokine storm is of priority to overcome the problem. We found that IL-33 and sST2 levels were higher in the plasma of HFRS patients, especially in their acute phase. Although both of them were positively correlated with the severity of the diseases, they acted in different roles in the regulation of the immune response during HTNV infection. *In vitro* study showed that IL-33 acted as an initiator of the cytokine storm in HTNV-infected endothelial cells, while sST2 acted as an inhibitor of the process. For the first time, we defined the IL-33/ST2 axis as inflammatory regulators during HTNV infection. Our results may provide a novel therapeutic target of HTNV infections.

## Introduction

Hantaan virus (HTNV) is a member of the *Bunyaviridae* family [1]. HTNV can cause severe lethal haemorrhagic fever with renal syndrome (HFRS) in humans, which is characterised by increased capillary permeability and thrombocytopenia. At present, the pathogenesis of HFRS remains unclear. Previous reports suggest that cytokine storm is a potential mechanism of HFRS pathogenesis [2]. Increased cytokines, such as IL-6, IL-8, and CXCL10, have been found in the serum, plasma, urine, and tissues of patients with hantavirus infections and correlate with the severity of the disease [3–7]. It has also been suggested that the viral infection of endothelial cells plays an important role in capillary leakage [8], which is triggered by cytotoxic CD8^+^ T cells and augmented by pro-inflammatory cytokines [2].

Interleukin-33 (IL-33), a new member of the IL-1 cytokine family, serves as a ligand for the ST2 receptor [9]. Recent studies have suggested that IL-33 is specifically released during necrotic cell death but is intracellular during apoptosis. Because of these properties, IL-33 is identified as an “alarmin” and is defined as a member of danger-associated molecular pattern (DAMP) molecule for alerting the immune system after infection or injury [10]. As a potent inducer of the T-helper 2 (Th2) immune response, IL-33 promotes the production of Th2-associated cytokines, such as IL-4, IL-5, and IL-13, mostly released from polarized Th2 cells [9]. In addition to Th2-related effects, IL-33 also induces inflammatory responses in endothelium [11] and epithelium [12].

The ST2 gene, a member of the IL-1RL1 superfamily, is known to encode at least 3 isoforms of ST2 proteins by alternative splicing: a membrane-anchored long form (ST2L), a secreted soluble form (sST2), and a membrane-anchored variant form (ST2V) [13–14]. sST2, serving as a decoy receptor for IL-33, can neutralize the function of IL-33. ST2L has been reported to be constitutively expressed by mast cells as well as Th2 cells. Upon binding with IL-33, ST2L forms a complex with the IL-1R accessory protein (IL-1RAcP), recruits the adaptor protein MyD88, activates MAP kinases (MAPK) and NF-κB pathways, and promotes the production of inflammatory mediators [9].

Numerous studies have reported the expression and function of IL-33/ST2 signalling in various diseases. IL-33/ST2 overstimulation has been implicated in airway inflammatory diseases [15–17], autoimmune diseases [18], viral infection diseases [19–20], and many other diseases [21–24], suggesting an important role for IL-33/ST2 in the development of inflammatory pathologies. However, the mechanism by which the IL-33/ST2 axis exerts its immunomodulatory effects in HFRS has not yet been elucidated.

In this study, we have quantified for the first time the plasma levels of IL-33 and sST2 in HFRS patients, analyzed the relationships between IL-33, sST2, and disease severity-indicating parameters in vivo, and explored the role of IL-33/ST2 in regulating immune response in vitro during HTNV infection. We found that elevated plasma IL-33 and sST2 levels were associated with the development of HFRS. Our in vitro examination indicated that IL-33 could enhance the production of pro-inflammatory cytokines in HTNV-infected endothelial cells and that this process could be inhibited by the recombinant sST2. Taken together, our data suggested that the IL-33/ST2 axis may function as an inflammatory regulator during HTNV infection.

## Materials and Methods

### Ethics Statement

The study was approved by the Institutional Review Board of the Fourth Military Medical University. Written informed consent was obtained directly from each subject for the collection of samples and subsequent analysis.

### Study Subjects and Sample Collection

Enrolled in the study were 23 hospitalised HFRS patients from Tangdu Hospital of the Fourth Military Medical University (Xi’an, China) from October 2012 to January 2013 (see Table 1). The clinical diagnosis of HFRS was confirmed by the detection of IgM antibodies against HTNV nucleocapsid protein. A total of 28 healthy donors were included as the normal control. The plasma samples were collected and stored as previously described [25–26]. Based on the classically defined 5 stages of HFRS, we classified the HFRS patients in this study into acute phase (including febrile, hypotensive, and oliguric stages) and convalescent phase (including diuretic and convalescent stages) [7,27–28]. The plasma viral load of the entire subjects group, an important indicator of disease severity, was determined using a quantitative 1-step real-time reverse-transcriptase polymerase chain reaction (RT-PCR) assay published previously [26].

**Table 1:** Characteristics of the HFRS patients at different stages of the disease.

	Acute	Convalescent
	Febrile/Hypotensive/Oliguric	Diuretic/Convalescent
Sample number	29	30
IL-33 (pg/ml)	68.0 (33.0–110.0)	19.5 (12.75–26.0)
sST2 (pg/ml)	2112.0 (639.0–7424.0)	129.0 (77.0–201.3)
White blood cell count (×10^3^/μl)	9.2 (7.2–14.2)	6.2 (5.4–7.5)
Platelet Count (×10^3^/μl)	31.0 (22.0–54.5)	196.0 (115.0–264.3)
blood urea nitrogen (μmol/L)	17.9 (8.0–22.2)	11.9 (6.1–15.5)
Serum Creatinine (μmol/L)	325.9 (118.1–588.0)	217.8 (98.5–404.5)
Viral load (log10 copies/ml)	6.1 (5.8–6.5)	5.9 (5.8–6.1)

Values represent medians with the corresponding interquartile range.

### Cells and Virus

Human umbilical vein endothelial cells (HUVECs) were prepared by a previously described method [29]. The HTNV strain 76–118 and inactivated HTNV (mock virus) control were prepared and stored in our lab as described [7]. For all infections, the virus was allowed to adsorb to HUVECs at a multiplicity of infection (MOI) of approximately 1 in serum-free EGM maintenance medium for 2 h at 37°C. The cells were then washed and incubated in EGM growth medium with 10% fetal bovine serum. The proportion of infected HUVECs was tested using immunofluorescence. At 48 hours postinfection, over 90% of the HUVECs expressed viral nucleocapsid protein in the cytoplasm.

HUVECs were seeded 24 h before treatment. When the cell confluence up to 60%-70%, the cells were infected with HTNV/mock virus (MOI = 1) for 48 h or treated with interleukin-33 (IL-33, R&D system, USA) for 6 h; alternatively, the cells were pre-infected with HTNV/mock for 48 h and then treated with 20 ng/ml IL-33 for another 6 h. To assess the role of ST2 and its signalling pathways in the induction of pro-inflammatory cytokines via IL-33 stimulation, HUVECs were exposed to human recombinant sST2 (R&D Systems, USA) or inhibitors (Calbiochem, USA) of the indicated signalling pathways at different concentrations for 2 h prior to the stimulation indicated above.

### ELISA

The amounts of IL-33 and sST2 present in HFRS patient plasma were determined using ELISA kits from eBioscience (USA) and RayBiotech (USA), respectively, according to each manufacturer’s instructions.

### RNA Interference

For the specific knockdown of ST2 and p65, double-stranded small interfering RNAs were synthesised by Gene Pharma (China) using the sequences shown in the supporting information (S1 Table). siRNA transfection into HUVECs was performed using Lipofectamine 3000 (Invitrogen, USA) according to the manufacturer’s protocol. The efficiency and confirmation of the knockdown was identified by determining the mRNA and protein levels of the target gene after transfection of the corresponding siRNA or the mock vector into HUVECs.

### RNA Extraction and Real-Time PCR

Total RNA from HUVECs was extracted using TRIzol (Invitrogen, USA) according to the manufacturer’s protocol, and 1 μg was used for cDNA synthesis (Takara, Japan). A quantitative analysis of mRNA expression was determined by quantitative real-time PCR using the SYBR Green (Takara, Japan) detection method. The specific primers for each gene are shown in the supporting information S2 Table. The reactions were analysed using a BIO-RAD system (CFX96 Real-Time System). The delta delta Ct method was used to calculate each gene of interest, and each gene was normalized to the housekeeping gene GAPDH and presented as copies of the normal medium control for HUVEC studies.

### Western Blot (WB) Analysis

The cells were exposed to virus or IL-33 for predetermined periods of time. The cells were then washed with PBS and lysed in RIPA buffer. For western blotting, 20 μg of total protein from each sample was subject to a stacking gel and separated by a 10% SDS-PAGE separating gel using the Tris-glycine system and then transferred onto nitrocellulose membranes (Millipore, USA). The membranes were blocked in 3% BSA and then probed overnight at 4°C with antibodies specific, respectively, for ST2 (Abcam, UK), p65, p-IKK, IKK, p-IκB, IκB, p-JNK, JNK, p-ErK, ErK, p-p38, p38, and β-tubulin (Cell Signaling Technology, USA), and GAPDH (Ambion, USA). The membranes were washed and incubated with an HRP-conjugated goat anti-mouse antibody or HRP-conjugated goat anti-rabbit antibody (Pierce, USA). After washing the membranes, the blots were developed using electrochemiluminescence (Alpha Innotech, USA).

### Flow Cytometry (FCM) Assay

HUVECs from the different treatment groups were collected and resuspended at a concentration of 10^7^cells/ml in flow buffer (PBS + 1% FCS + 0.1% NaN_3_). Fc receptors on the HUVECs were blocked by the addition of normal goat serum. Then, 10^6^ cells were incubated for 30 minutes at 4°C with the anti-ST2 monoclonal antibody (Abcam, UK). After washing the cells twice with flow buffer, the cells were stained with phycoerythrin (PE)-conjugated goat anti-mouse secondary antibody (BD Biosciences, USA) and with an isotype antibody as a negative control. The cells were washed twice with flow buffer, and 200 μL of 4% formalin was added to fix the cells. A minimum of 100,000 cells were acquired using a BD FACS Calibur Flow Cytometer (Beckman Coulter, USA) and analysed using Flowjo software (Treestar, USA).

### Dual Luciferase Assays

The pNF-κB-luc plasmid was purchased from Beyotime, China. HUVECs were seeded in a 24-well plate. When the confluence was approximately 70%, the cells were transfected with 1.6 μg/well of pNF-κB-luc plasmid and 5 ng/well of the pRL-TK plasmid using jetPEI-HUVEC Polyplus Transfection reagent (Polyplus, USA) according to the manufacturer’s protocol. After 4 hours, the HUVECs were infected with HTNV (MOI = 1) for an additional 48 h and then treated with 20 ng/ml IL-33 for 6 h. The luciferase activity in each sample was then detected using the Dual-luciferase reporter assay system (Promega, USA) according to the manufacturer’s instructions, and the transfection efficiency was normalised to the Renilla luciferase activity. For data analyses, the medium control was set as 1.

### Statistical Analysis

The analysis was performed using SPSS and GraphPad Prism5 software. The statistical significance was determined using one-way ANOVA. The Spearman correlation test was used to test the correlation between the IL-33/sST2 concentrations and clinical parameters. A *p* value less than 0.05 was considered to be statistically significant.

## Results

### Elevated IL-33 and sST2 Levels in HFRS Patients’ Plasma Are Positively Correlated with HFRS Severity

A total of 23 HFRS patients with 59 plasma samples were collected at the acute (febrile/hypotensive/oliguric), or convalescent (diuretic/convalescent) phases of the disease. Each patient contained two, three, or four samples collected in different stages. The details of the clinical parameters detected during the hospitalization of the patients are summarized in Table 1.

**Fig 1:**
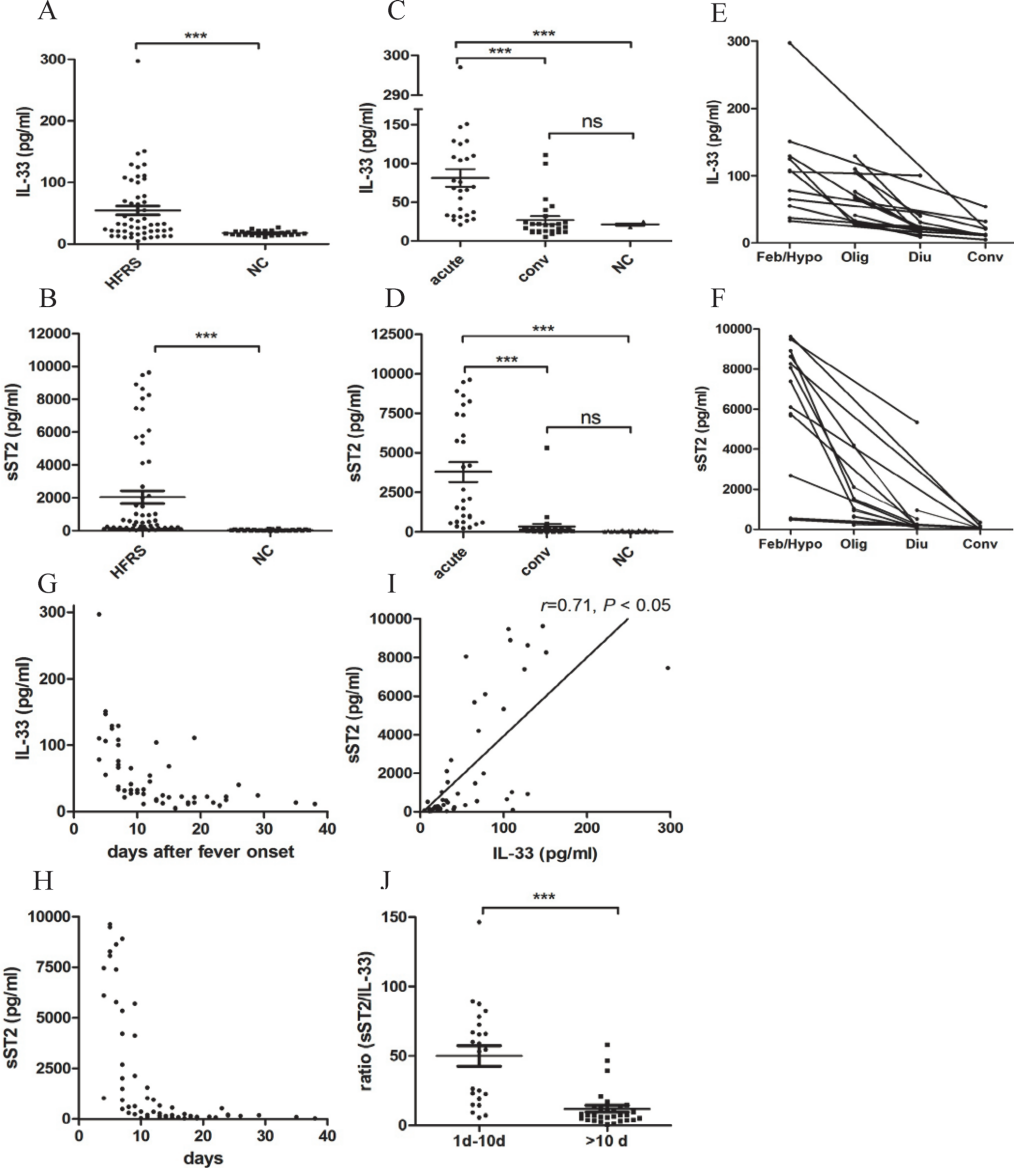
Increased IL-33 and sST2 levels in HFRS patients’ plasma. Scatter diagram displaying the protein levels of IL-33 and sST2 in the plasma of HFRS patients. Comparison of plasma IL-33 (A) and sST2 (B) contents between HFRS patients and healthy donors (NC). Contents of IL-33 (C) or sST2 (D) in the acute phase of HFRS (including febrile, hypotensive, or oliguric stage), the convalescent phase of HFRS (including diuretic or convalescent stage), and healthy donors (NC). Data are the means±SE (HFRS, n = 59; NC, n = 28), ***p < 0.001, HFRS patients versus NC or acute phase versus convalescent phase and NC. Kinetic trends of IL-33 (E) and sST2 (F) levels in each HFRS patient. Kinetic analysis of plasma IL-33 (G) and sST2 (H) levels. Spearman correlation test showing that the plasma contents of sST2 and IL-33 in the entire group of subjects are positively correlated (I). The *r* and *p* values are indicated in the graphs. The 1–10 days group had higher ratios of sST2 to IL-33 than the > 10 days group (***p < 0.001) (J).

The mean levels of IL-33 (Fig. 1A) and sST2 (Fig. 1B) in the HFRS patients were respectively, 3 times or 38 times higher than that in the normal control. The IL-33 (Fig. 1C) and sST2 (Fig. 1D) contents in HFRS patients in the acute phase were both markedly higher than those in the convalescent phase and in the normal controls (*p* < 0.001). However, there was no significant difference in IL-33 or sST2 level between the convalescent-phase HFRS patients and the healthy donors (Fig. 1C-D). The individuals’ kinetic data was shown to determine the changes trends of IL-33 (Fig. 1E) and sST2 (Fig. 1F) levels in each HFRS patient, who expressed much higher levels of IL-33 and sST2 in the early phase. Generally, the level of IL-33 peaked (297.00 pg/ml) in the early phase of HFRS and decreased sharply at 10 days after fever onset (Fig. 1G). Although the kinetic changes in sST2 were similar to that of IL-33, the decrease of sST2 was much more dramatic than that of IL-33 (Fig. 1H). Interestingly, the plasma levels of IL33 and sST2 were also positively correlated, as determined by the Spearman correlation analysis (*r* = 0.71, *p* < 0.05) (Fig. 1I). Within the first 10 days of fever onset, the ratio of sST2 to IL-33 was 4.70 times higher than that after 10 days of fever (Fig. 1J).

**Fig 2:**
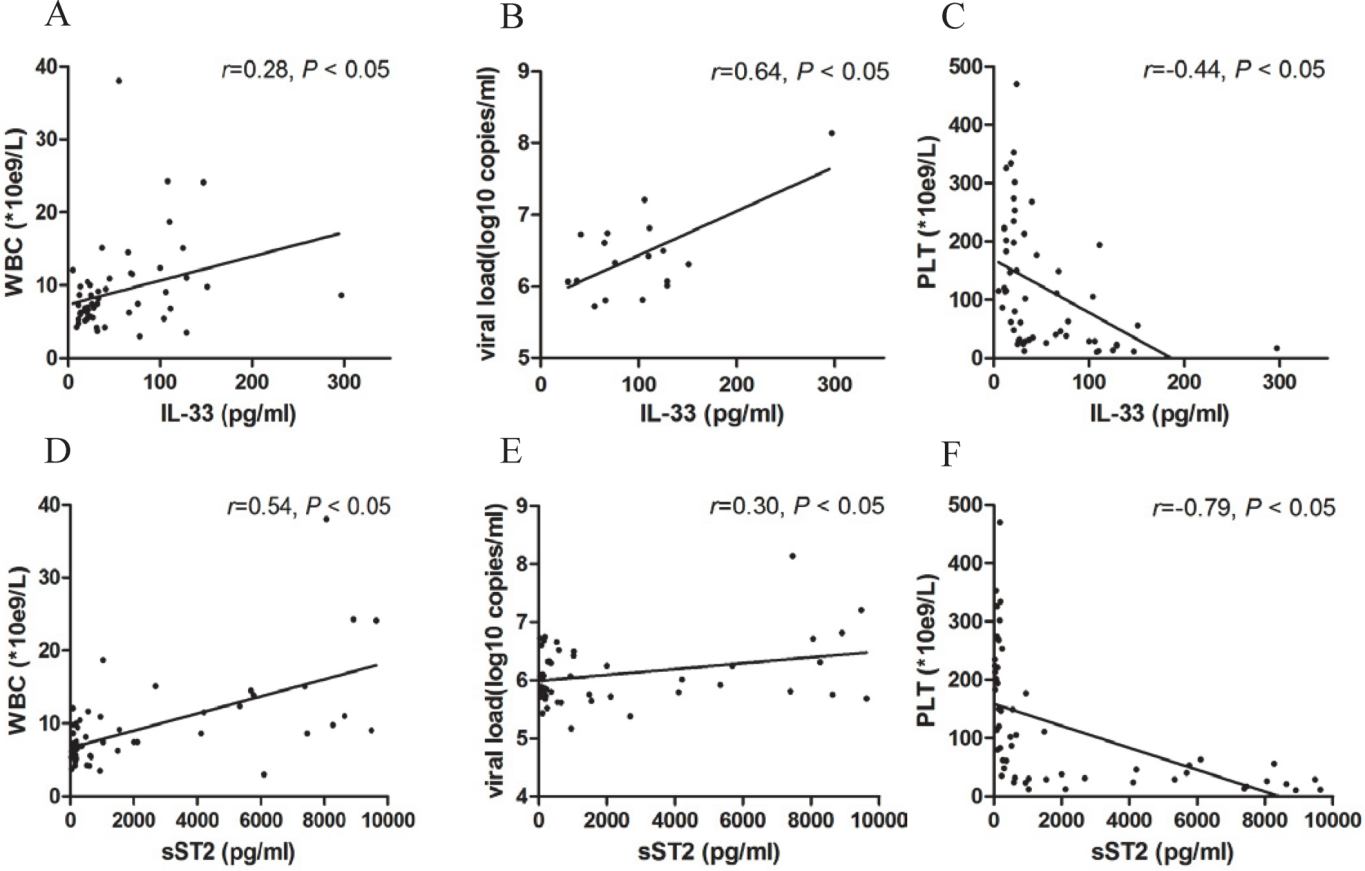
Increased levels of IL-33 and sST2 in HFRS patients’ plasma are positively correlated with HFRS severity. Spearman correlation test representing that the plasma IL-33 contents in the entire group of subjects are positively correlated with the white blood cell (WBC) count (A) and viral load (B) but negatively correlated with the platelet count (PLT) (C). The plasma sST2 contents in the entire group of subjects are also positively correlated with the white blood cell (WBC) count (D) and viral load (E) but negatively correlated with the platelet count (PLT) (F). The *r* and *p* values are indicated in the graphs.

Spearman correlation analysis revealed that this increasing level of IL-33 was correlated with increasing WBC count (*r* = 0.28, *p* < 0.05) (Fig. 2A) and viral load (*r* = 0.64, *p* < 0.05) (Fig. 2B) and decreasing PLT count (*r* = −0.44, *p* < 0.05) in HFRS patients (Fig. 2C). Similarly, the sST2 content was correlated with increasing WBC counts (*r* = 0.54, *p* < 0.05) (Fig. 2D) and viral load (*r* = 0.64, *p* < 0.05) (Fig. 2E) and decreasing PLT counts (*r* = −0.79, *p* < 0.05) in HFRS patients (Fig. 2F).

### IL-33 Promotes Pro-inflammatory Cytokine Production by HTNV-infected HUVECs

**Fig 3:**
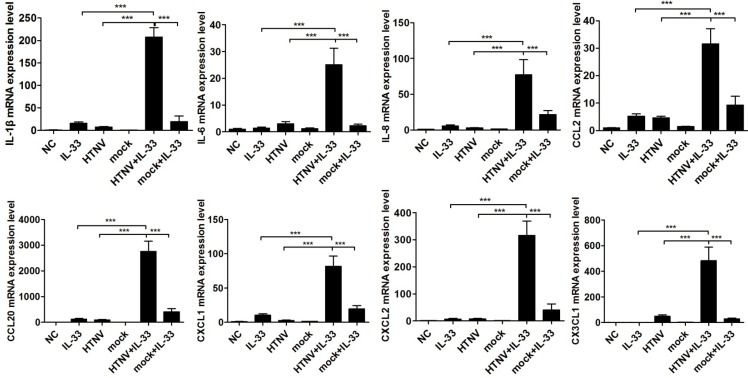
IL-33 induces the expression of pro-inflammatory cytokines in HTNV-infected HUVECs. HUVECs were infected with HTNV/mock virus (MOI = 1) for 48 h or stimulated with IL-33 (20 ng/ml) for 6 h or first infected with virus for 48 h and then treated with IL-33 (20 ng/ml) for another 6 h. The mRNA expression of IL-1β, IL-6, IL-8, CCL2, CCL20, CXCL1, CXCL2, and CX3CL1 was determined by real-time PCR. Untreated HUVECs were set as the normal control (NC). Data are shown as the mean ± SD of triplicate samples and are representative of experiments with three independent HUVEC donors, *p < 0.05, **p < 0.01, ***p < 0.001.

To elucidate the role of IL-33 in modulating the pattern of cytokine production, we evaluated the mRNA expression of pro-inflammatory cytokines and chemokines in primary HUVECs by real-time PCR. Using cells only treated with IL-33 or cells only infected with HTNV as controls, the mRNA expression of IL-1β, IL-6, IL-8, CCL2, CCL20, CXCL1, CXCL2, and CX3CL1 was significantly induced in HUVECs prior to infection with HTNV (MOI = 1) for 48 h and then exposed to IL-33 (20 ng/ml) for 6 h (Fig. 3). The cells pre-infected with inactive HTNV (mock virus) for 48 h did not show the same effect (Fig. 3). Because previous reports have demonstrated that IL-33 could potently induce the production of Th2-associated cytokines [9], we also measured the mRNA levels of IL-4, IL-5, and IL-13 and found no inducement in our experimental system (S1 Fig.).

### IL-33 Mediates Inflammatory Responses via the ST2 Receptor in HUVECs

**Fig 4:**
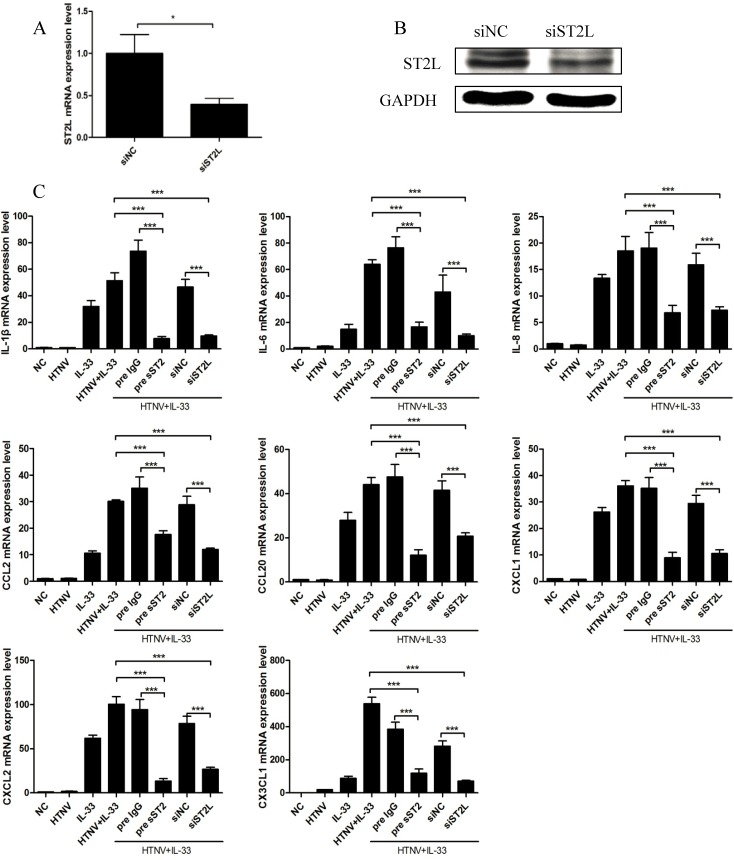
IL-33 mediates inflammatory responses via the ST2 receptor in HTNV-infected HUVECs. HUVECs were transfected with an siRNA specific to ST2L for 6 h and then infected with HTNV (MOI = 1) for 48 h. The cells were harvested being treated with 20 ng/ml IL-33 for another 6 hours. The mRNA (A) and protein levels (B) of ST2L were reduced when compared to the scramble control. *p < 0.05, siST2 versus nontargeting control siNC. (C) HUVECs were transfected with an siRNA specific to ST2L for 6 h or first prior incubated with soluble recombinant human ST2 protein (sST2, 100 ng/ml) for 2 h; siNC or isotype IgG (100 ng/ml) was set as the control, respectively. The treated cells were then stimulated with both HTNV and IL-33, as indicated above. The mRNA levels of IL-1β, IL-6, IL-8, CCL2, CCL20, CXCL1, CXCL2, and CX3CL1 were determined by real-time PCR. The results shown are the mean± SD of triplicate samples and are representative of experiments with three independent HUVEC donors, ***p < 0.001.

We investigated whether the ST2 signalling pathway participates in IL-33-mediated inflammatory responses in HUVECs infected with HTNV. Using a non-targeting siRNA as the scramble control, HUVECs were transfected with an siRNA against ST2L for 6 h, and the cells were treated with both HTNV and IL-33, as indicated above. The mRNA and protein levels of ST2L were determined by real-time PCR (Fig. 4A) and western blotting (Fig. 4B), respectively. The induction of pro-inflammatory cytokines by both HTNV infection and IL-33 treatment was significantly inhibited when ST2L was depleted (Fig. 4C). Pre-incubation of HUVECs with soluble recombinant human ST2 protein (100 ng/ml) two hours prior to HTNV infection and IL-33 stimulation, with the recombinant human IgG (100 ng/ml) as the isotype control, resulted in the significant suppression of the production of these pro-inflammatory cytokines and chemokines (Fig. 4C). These results suggested that an ST2-dependent pathway is involved in IL-33-mediated inflammatory responses in HUVECs.

**Fig 5:**
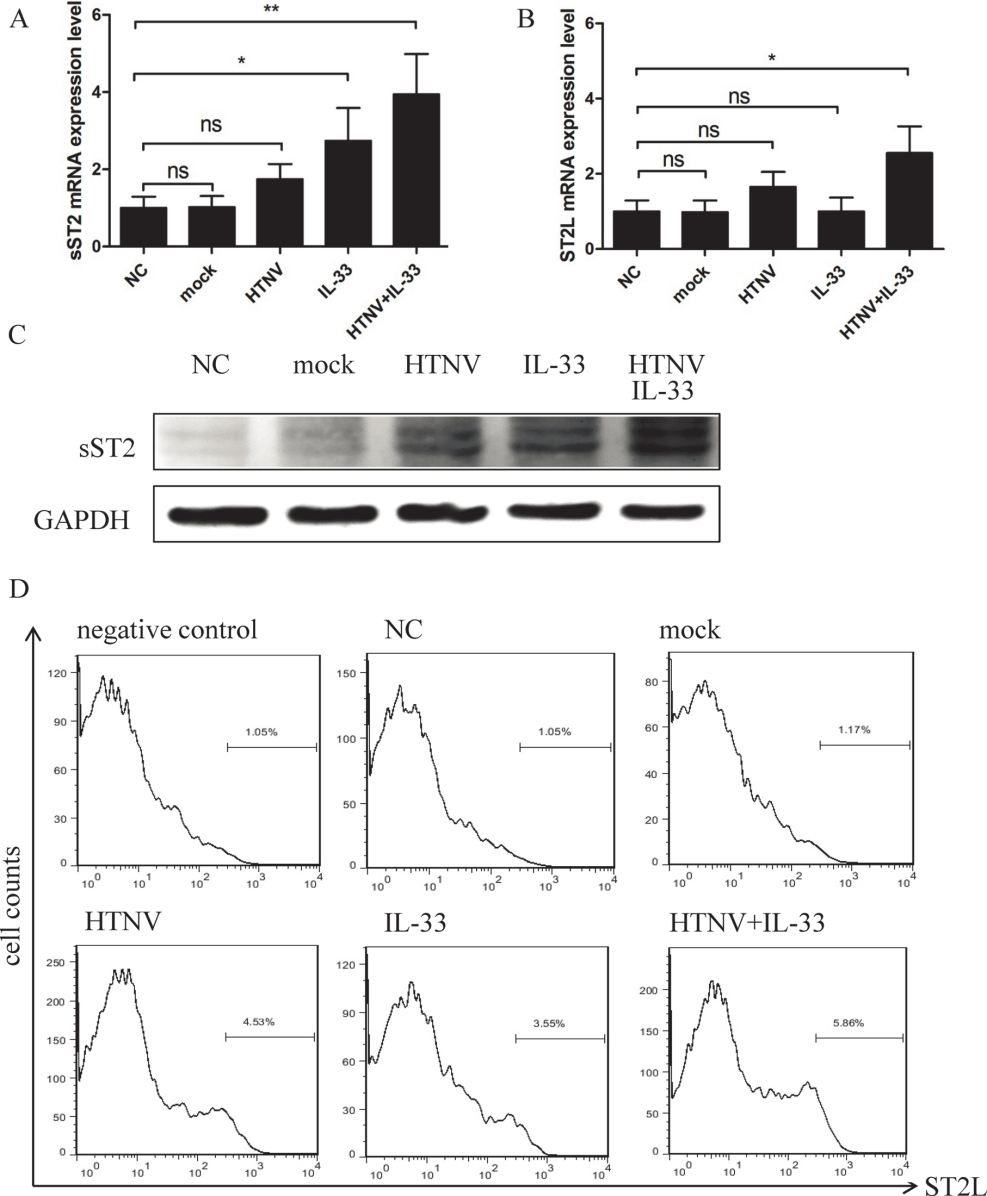
Expressions of sST2 and ST2L in HUVECs. HUVECs were treated as previously indicated, and whole-cell lysates were harvested. The mRNA levels of sST2 (A) and ST2L (B) were determined by real-time PCR. The protein levels of sST2 and GAPDH were analysed by western blotting (C), and the level of ST2L expression was determined by flow cytometric analysis (D). Data are the means ± SE, *p < 0.05, **p < 0.01.

Next, we measured the mRNA and protein levels of both sST2 and ST2L in HUVECs. The HUVECs stimulated with both HTNV and IL-33 expressed higher levels of sST2 and ST2L, both at the mRNA (Fig. 5A-B) and protein levels (Fig. 5C-D). Our findings suggested that HTNV and IL-33 could synergistically promote the induction of ST2 in HUVECs.

### IL-33 Enhances the Phosphorylation of NF-κB in HTNV-infected HUVECs

**Fig 6:**
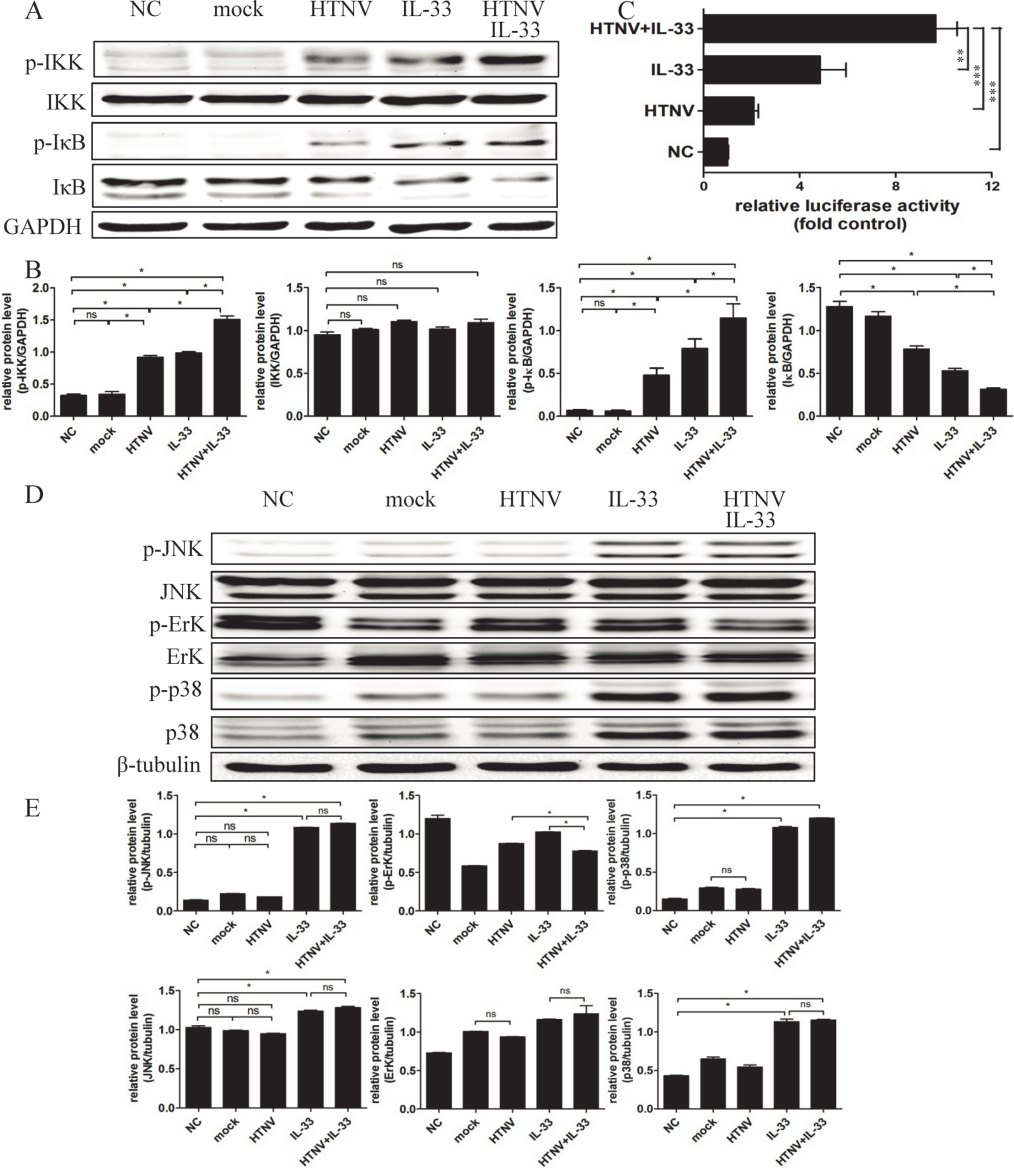
IL-33 and HTNV synergistically induce the activation of the NF-κB pathway. (A) HUVECs were infected with HTNV/mock virus (MOI = 1) for 48 h or were stimulated with IL-33 (20 ng/ml) for 8 min or were pre-infected with HTNV for 48 h and treated with IL-33 (20 ng/ml) for another 8 min. Whole-cell lysates were harvested, and the amounts of phospho-IKK, total IKK, phospho-IκB, total IκB, and GAPDH were determined by western blotting. (B) A semiquantitative analysis of p-IKK, IKK, p-IκB, and IκB levels was performed using Image J software. The expression of GAPDH was used as the control. (C) The plasmid pNF-κB-luc was transfected into HUVECs. Four hours after transfection, the cells were infected with HTNV for 48 h or were treated with IL-33 (20 ng/ml) for 6 h or cells were first infected with HTNV for 48 h and then treated with IL-33 (20 ng/ml) for another 6 h. HUVECs treated only with growth medium were set as the normal control (NC). The relative luciferase activities were detected. NC is set as one, and the others are presented as fold values relative to NC. (D) The phospho-JNK, total JNK, phospho-ErK, total ErK, phospho-p38, total p38, and tubulin levels were determined by western blotting using the same cell lysates indicated above. (E) A semiquantitative analysis of the p-JNK, JNK, p-ErK, ErK, p-p38, and p38 levels was performed using Image J software. The expression of β-tubulin was set as the control. Data are the means ± SE, *p < 0.05, **p < 0.01, ***p < 0.001.

We then investigated the signalling pathways involved in the IL-33-stimulated inflammatory response in HUVECs pre-infected with HTNV. Although the signalling pathways activated by IL-33 remain poorly understood, it has been reported that IL-33 could active the NF-κB and MAPK pathways [9]. Therefore, we performed western blotting and found that the phosphorylation of both IKK and IκB was increased in the HUVECs infected with HTNV alone for 48 h and in the HUVECs only treated with IL-33 (20 ng/ml) for 8 min. Interestingly, the levels of p-IKK and p-IκB were notably enhanced when the HUVECs were pre-infected with HTNV for 48 h and then treated with IL-33 (20 ng/ml) for 8 min together (Fig. 6A). Although the total amount of IKK protein was unchanged in the differently treated HUVECs, the total amount of IκB proteins was reduced, whereas the phosphorylation form increased (Fig. 6A). The densitometric analysis of the WB results in Fig. 6B shows the ratios of the protein levels of IKK and IκB relative to the loading control GAPDH. To further confirm the activation of the NF-κB pathway, a dual luciferase assay was performed by transfecting the pNF-κB-luc plasmid into HUVECs. The luciferase activity indicated that the activation of the NF-κB pathway was enhanced in the HUVECs treated with both HTNV and IL-33 (Fig. 6C). We also conducted western blotting to evaluate whether the MAPK pathway is involved in the IL-33 responses in HUVECs and found that the expression of p-JNK, p-ErK, and p-p38 were not enhanced when IL-33 was added in together with HTNV (Fig. 6D). The ratios of the protein levels of JNK, ErK, and p38 relative to the loading control β-tubulin were revealed by the densitometric analysis of the western blot results (Fig. 6E). Our findings indicated that HTNV and IL-33 treatment enhanced the activation of the NF-κB pathway rather than the MAPK pathway.

**Fig 7:**
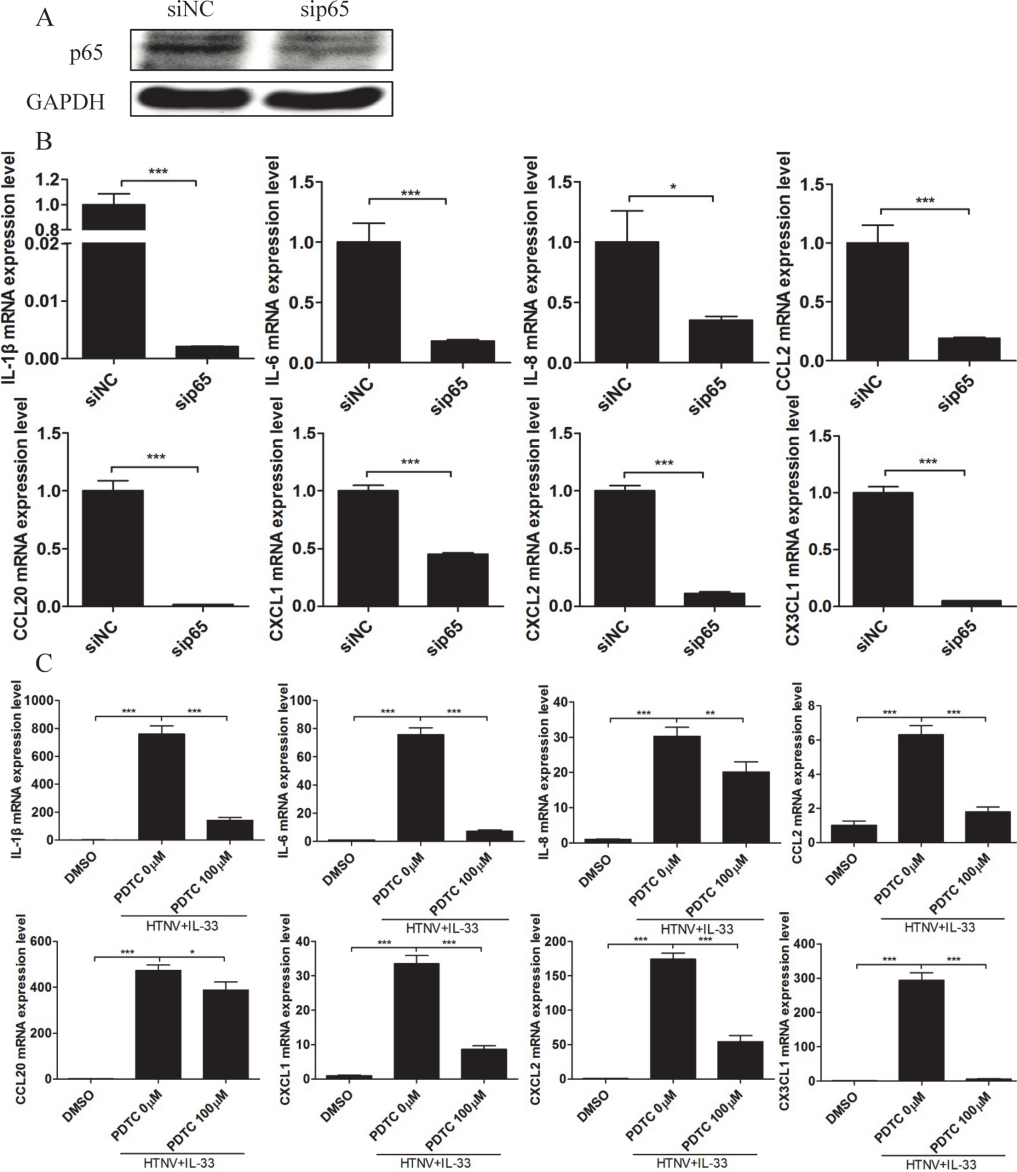
Divergent effects of sip65 and PDTC on IL-33-mediated inflammatory responses in HUVECs infected with HTNV. (A) An siRNA specific to p65 was transfected into HUVECs for 6 h, and the cells were treated as indicated above. Half of the cell lysate was collected, and the expression of p65 and GAPDH was analysed by western blotting. (B) RNA was extracted from the other half, and the mRNA levels of pro-inflammatory cytokines were determined by real-time PCR. All the experiments were repeated in triplicate. The mRNA data were generated from three independent experiments using three independent HUVECs donors. (C) HUVECs were first exposed to PDTC (100 μM) for 2 h and infected with HTNV (MOI = 1) for 48 h and treated with IL-33 (20 ng/ml) for another 6 h. Cells in a medium containing DMSO were set as the reagent control. RNA was extracted from these cells, and the mRNA levels of pro-inflammatory cytokines were determined by real-time PCR. All the experiments were repeated in triplicate. The mRNA data were generated from three independent experiments using three independent HUVECs donors. Data are the means ±SE. *p < 0.05, **p < 0.01, ***p < 0.001, siRNA specific to p65 versus siNC-treated cells, PDTC 0 μM versus PDTC 100 μM, or PDTC 0 μM versus the DMSO control.

To further verify the role of the NF-κB pathway in the IL-33-mediated inflammatory response, we transfected an siRNA specific to the p65 subunit into HUVECs and demonstrated that the knockdown of p65 (Fig. 7A) could significantly impair the production of pro-inflammatory cytokines in HUVECs treated with both HTNV and IL-33 (Fig. 7B). Additionally, pre-treatment of the NF-κB activation inhibitor pyrrolidine dithiocarbamic acid (PDTC, 100 μM) markedly suppressed the mRNA expression of these pro-inflammatory cytokines (Fig. 7C). These findings suggested that the NF-κB pathway plays an important role in the regulation of HTNV/IL-33-mediated inflammatory responses in HUVECs.

## Discussion

Our study demonstrated that the higher plasma IL-33 and sST2 levels in the HFRS patients, which were associated with the disease severity-indicating clinical parameters, may exert their pro- and anti- functions in inflammatory response during HTNV infection, respectively.

As an alarmin, IL-33 is mainly produced by structural and lining cells, such as endothelial cells, fibroblasts, and epithelial cells, where the first line of host defence against pathogens normally arises [30]. In our study, elevated IL-33 was detected in the plasma of HFRS patients, indicating that HTNV infection might induce cellular damage or necrosis (Fig. 1A). To identify the source of this high level of IL-33 during HTNV infection, we measured IL-33 in HTNV-infected HUVECs. However, we did not detect IL-33 at either the mRNA or protein level (S2 Fig.). Thus, we hypothesised that under in vivo conditions, endothelial cells, epithelial cells, and fibroblasts may undergo necrosis and release IL-33 during HTNV infection.

The roles of IL-33 in various diseases have been discussed recently. In mice infected with influenza virus, IL-33 treatment led to significantly reduced inflammation and pathology of the lungs [30]. IL-33 also directly drives protective antiviral CD8^+^ T cell responses against lymphocytic choriomeningitis virus (LCMV) infection in mice [31]. These results present an important protective role of IL-33 in some infectious diseases. However, in Th2-mediated inflammatory diseases, such as asthma, rheumatological diseases, and inflammatory skin disorders, IL-33 appears to have pro-inflammatory effects and exacerbates the diseases [32]. Hantavirus pathology is suggested to be linked to T cell activation, either through the excess secretion of pro-inflammatory cytokines or through the CTL-mediated killing of infected cells [33]. It was also demonstrated that Th1 and CTL subsets, rather than the Th2 subset, preferentially proliferate and differentiate during the course of HFRS [34]. Therefore, whether IL-33 plays a protective role or a damaging role during HTNV infection remains an enigma. In our study, we found that elevated IL-33 was positively correlated with the severity of HFRS (Fig. 2A-C). Our in vitro results suggested that in HTNV-pre-infected HUVECs, IL-33 significantly enhanced the production of pro-inflammatory cytokines (Fig. 3), rather than the Th2-related cytokines, such as IL-4, IL-5 and IL-13 (S1 Fig.). Our in vivo study also showed that pro-inflammatory cytokines like IL-6 and IL-8 were elevated in the same HFRS patients’ plasma, especially in their acute phases (S3 Fig.). However, the plasma levels of IL-4, IL-5, and IL-13 cannot be detected. Combined with previous reports that IL-33 could increase vascular permeability in HUVECs [35], we believe that IL-33 may act as an initiator of the “cytokine storm” and contribute to the development of HFRS. However, the role of IL-33 in the regulation of Th1 cells, Th2 cells, NK cells, and mast cells during HTNV infection still needs to be investigated.

The ST2/IL-33 signalling pathway has been reported to participate in the pathophysiology of numerous inflammatory and immune diseases [18–20]. To our knowledge, this is the first study to measure plasma IL-33 and sST2 levels simultaneously in patients with HFRS and discuss their roles during HTNV infection. Our results indicated that high levels of IL-33 and sST2 could act as biomarkers of HFRS development (Fig. 2). It has been reported that sST2 has immunosuppressive activity and direct anti-inflammation action [36]. In our study, recombinant sST2 could inhibit the IL-33-induced inflammatory response (Fig. 4C). To exert its regulatory function effectively, sST2 could be actively secreted by HUVECs stimulated by both HTNV and IL-33 (Fig. 5A), which was a more rapid route than sST2 shedding from ST2L, the extracellular domain of which shares a common sequence with sST2 [37]. Therefore, the increased expression of sST2 in HFRS plasma could be a physiological natural response mechanism for suppressing the damaging inflammatory responses induced by IL-33, preventing further ST2L-mediated immune cell activation and actively participating in the regulation of the immune system. As shown in Fig. 1J, during the first 10 days of disease onset, the sST2 content was more than 50 times the IL-33 content to balance the IL-33-mediated inflammatory response. Although we cannot at present affirm the protective effect of sST2 during HTNV infection in vivo, because of the lack of proper animal models for HFRS [38], our results add weight to understanding the important role of IL-33/ST2 axis in the regulation of “cytokine storm” during the acute phase of HFRS and provide a possible therapeutic target of the HFRS.

An important consideration arising from this study is why in HUVEC model, HTNV infection or IL-33 treatment alone did not induce excess cytokine production, whereas their combination augmented the expression of pro-inflammatory cytokines. IL-33 is a selective activator and preferentially targets nonquiescent HUVECs [11]. Previous reports have demonstrated that HTNV infection could induce increased levels of VCAM-1 and ICAM-1 in HUVECs [39], which could drive HUVECs into the nonquiescent state. Therefore, we hypothesised that HTNV infection activated HUVECs, causing them to become nonquiescent cells, which then became the target cells for IL-33. Thus, IL-33 could augment its pro-inflammatory function. Furthermore, HTNV infection could activate the NF-κB pathway in HUVECs [7,39]. Acting as cross-talk between HTNV and IL-33, the NF-κB pathway was highly activated when the HUVECs were treated with both HTNV and IL-33 (Fig. 6A-C). Taken together, nonquiescence and the enhanced activation of the NF-κB pathway may contribute to the synergistic effect on cytokine production induced by both HTNV and IL-33 in HUVECs.

Different infectious diseases may have different cytokine expression profiles. The mechanisms of initiating and fading of the “cytokine storm” may be also various in each infectious disease. Although Peng et.al have showed that during *Angiostrongylus cantonensis* infection, both splenocytes and brain mononuclear cells became IL-33 responsive and produced IL-5 and IL-13 [21], we are still lack of knowledge whether the mechanisms showed in our study are specific for HFRS. At present, we cannot demonstrate these mechanisms on other infectious diseases for the lack of other pathogens in our lab. Further studies are still needed to address this issue.

Overall, our results indicate that the IL-33/ST2 axis, serving as an important regulator of the inflammatory response during HTNV infection, may be involved in the pathogenesis of HFRS. The utilisation of sST2 to selectively reduce IL-33/ST2, with a consequent decrease in the inflammatory response in endothelial cells, may be exploited as a therapeutic target for Hantavirus infections.

## Supporting Information

S1 Table Specific siRNA for use in RNA interference. (DOC)

S2 Table Primer sequences for use in real-time PCR. (DOC)

S1 Fig IL-33 cannot induce the expression of IL-4, IL-5, and IL-13 in HTNV-infected HUVECs. HUVECs were infected with HTNV/mock virus (MOI = 1) for 48 h or stimulated with IL-33 (20 ng/ml) for 6 h or first infected with virus for 48 h and then treated with IL-33 (20 ng/ml) for another 6 h. The mRNA expression of IL-4, IL-5, and IL-13 was determined by real-time PCR. Untreated HUVECs were set as the normal control (NC). Data are shown as the mean ± SD of triplicate samples and are representative of experiments with three independent HUVEC donors. NA: none of any.
(TIF)

S2 Fig HTNV infection cannot induce the expression of IL-33. HUVECs were infected with HTNV (MOI = 1) for 0 h, 24 h, or 48 h, respectively. The mRNA level of IL-33 was determined by real-time PCR (A). The protein level of IL-33 was detected in the supernatant of the HUVECs by ELISA (B). NA: none of any.
(TIF)

S3 Fig Increased IL-6 and IL-8 levels in HFRS patients’ plasma. Scatter diagram displaying the protein levels of IL-6 and IL-8 in the plasma of HFRS patients detected by the ELISA kits (eBioscience, USA). Comparison of plasma IL-6 (A) and IL-8 (C) contents between HFRS patients and healthy donors (NC). Contents of IL-6 (B) or IL-8 (D) in the acute phase of HFRS (including febrile, hypotensive, or oliguric stage), the convalescent phase of HFRS (including diuretic or convalescent stage), and healthy donors (NC). Data are the means±SE (for IL-6, HFRS, n = 34; NC, n = 9; for IL-8, HFRS, n = 58; NC, n = 9), *p < 0.05, ***p < 0.001, HFRS patients versus NC or acute phase versus convalescent phase and NC.
(TIF)
