# Supplementary material for: IL-33/ST2 Correlates with Severity of Haemorrhagic Fever with Renal Syndrome and Regulates the Inflammatory Response in Hantaan Virus-Infected Endothelial Cells
Source: PLoS Negl Trop Dis. 2015 Feb 6;9(2):e0003514. doi: 10.1371/journal.pntd.0003514 (PMC4319827; doi:10.1371/journal.pntd.0003514)
Supplement: S1 Table — (DOC) [file pntd.0003514.s001.doc]

**S1 Table**. Specific siRNA for use in RNA interference

| Target gene | Sense (5’-3’) | Antisense (5’-3’) |
| --- | --- | --- |
| siST2L | CGAAAGAGCAGGCGGCACATT | AAUGUGCCGCCUGCUCUUUCG |
| sip65 | GCCUUAAUAGUAGGGUAAGTT | AACUUACCCUACUAUUAAGTG |
| siNC | UUCUCCGAACGUGUCACGUTT | ACGUGACACGUUCGGAGAATT |
