# Supplementary material for: IL-33/ST2 Correlates with Severity of Haemorrhagic Fever with Renal Syndrome and Regulates the Inflammatory Response in Hantaan Virus-Infected Endothelial Cells
Source: PLoS Negl Trop Dis. 2015 Feb 6;9(2):e0003514. doi: 10.1371/journal.pntd.0003514 (PMC4319827; doi:10.1371/journal.pntd.0003514)
Supplement: S2 Table — (DOC) [file pntd.0003514.s002.doc]

**S2 Table. Primer sequences for use in r**eal-time PCR

| Gene | Forward primers (5’-3’) | Backward primers (5’-3’) |
| --- | --- | --- |
| IL-1β | CTGTCCTGCGTGTTGAAAGA | TTGGGTAATTTTTGGGATCTACA |
| IL-4 | GCAGTTCTACAGCCACCA | GATCGTCTTTAGCCTTTCC |
| IL-5 | TCTACTCATCGAACTCTGCTGA | CCCTTGCACAGTTTGACTCTC |
| IL-6 | AGTGAGGAACAAGCCAGAGC | GTCAGGGGTGGTTATTGC |
| IL-8 | CTCTTGGCAGCCTTCCTGAT | TATGCACTGACATCTAAGTTCTTTAGCA |
| IL-13  IL-33 | GCATGGTATGGAGCATCAA  ACACGAGGAAGTGAAGTA | AGCATCCTCTGGGTCTTCT  TAGGCTCTGGTAGGTTAG |
| CCL2 | TCTGTGCCTGCTGCTCATAG | CAGATCTCCTTGGCCACAAT |
| CCL20 | GCTCCTGGCTGCTTTGATG | CAAAGTTGCTTGCTGCTTCTGA |
| CXCL1 | CCCCAAGAACATCCAAAGTG | GATGCAGGATTGAGGCAAG |
| CXCL2 | CTGCGCTGCCAGTGCTTG | CGATGCGGGGTTGAGACA |
| CX3CL1 | GAGCCGACTCCTTCTTCCC | CCCTCCATCCTGAGCCTTT |
| sST2 | CTGTCTGGCCCTGAATTTGC | TGGAACCACACTCCATTCTGC |
| ST2L | CTGTCTGGCCCTGAATTTGC | AGCAGAGTGGCCTCAATCCA |
| GAPDH | GACCTGACCTGCCGTCTA | AGGAGTGGGTGTCGCTGT |
