# Supplementary figures and images for: IL-33/ST2 Correlates with Severity of Haemorrhagic Fever with Renal Syndrome and Regulates the Inflammatory Response in Hantaan Virus-Infected Endothelial Cells
Source: PLoS Negl Trop Dis. 2015 Feb 6;9(2):e0003514. doi: 10.1371/journal.pntd.0003514 (PMC4319827; doi:10.1371/journal.pntd.0003514)

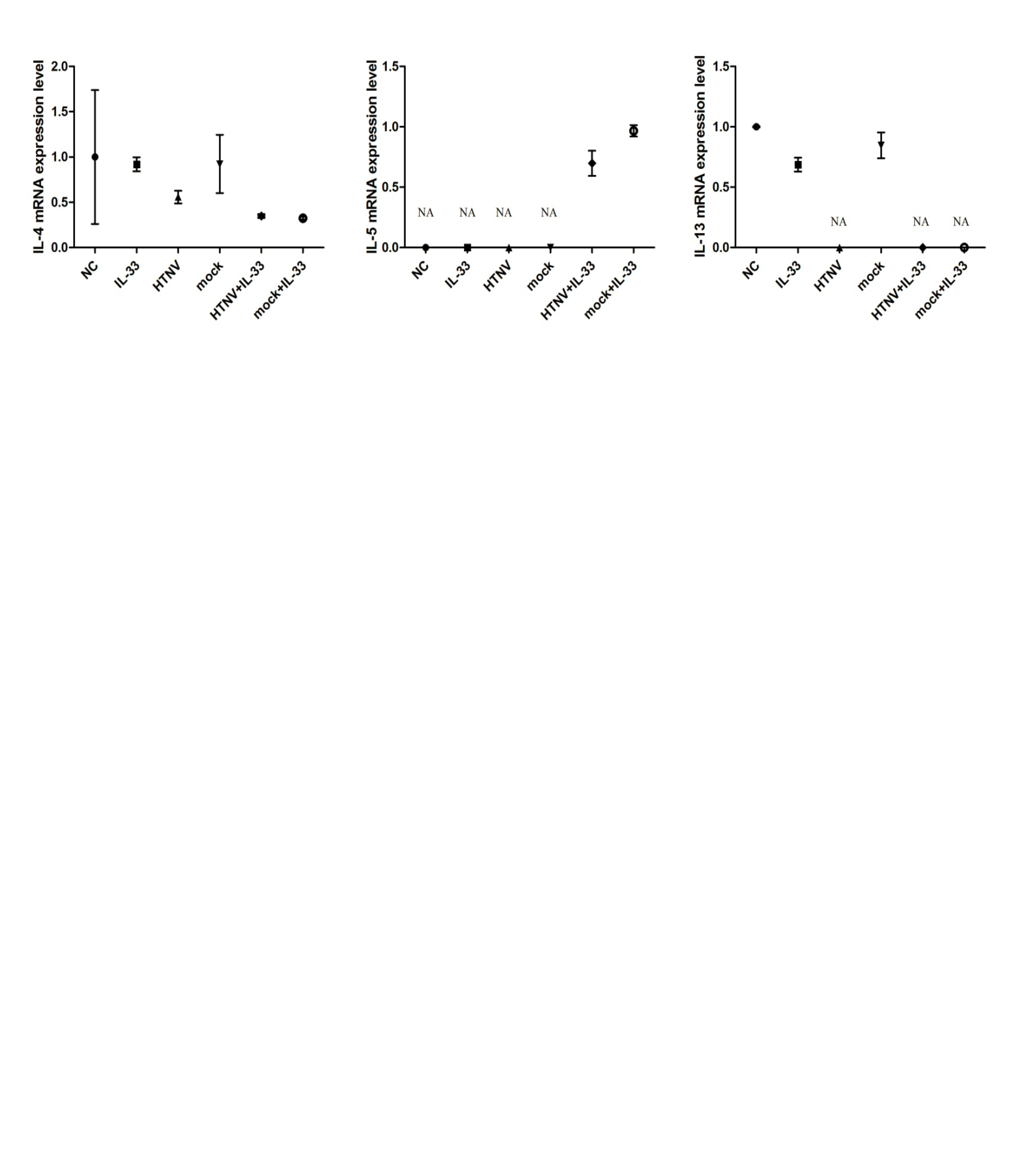

Supplement: S1 Fig — HUVECs were infected with HTNV/mock virus (MOI = 1) for 48 h or stimulated with IL-33 (20 ng/ml) for 6 h or first infected with virus for 48 h and then treated with IL-33 (20 ng/ml) for another 6 h. The mRNA expression of IL-4, IL-5, and IL-13 was determined by real-time PCR. Untreated HUVECs were set as the normal control (NC). Data are shown as the mean ± SD of triplicate samples and are representative of experiments with three independent HUVEC donors. NA: none of any. (TIF) [file pntd.0003514.s003.tif]

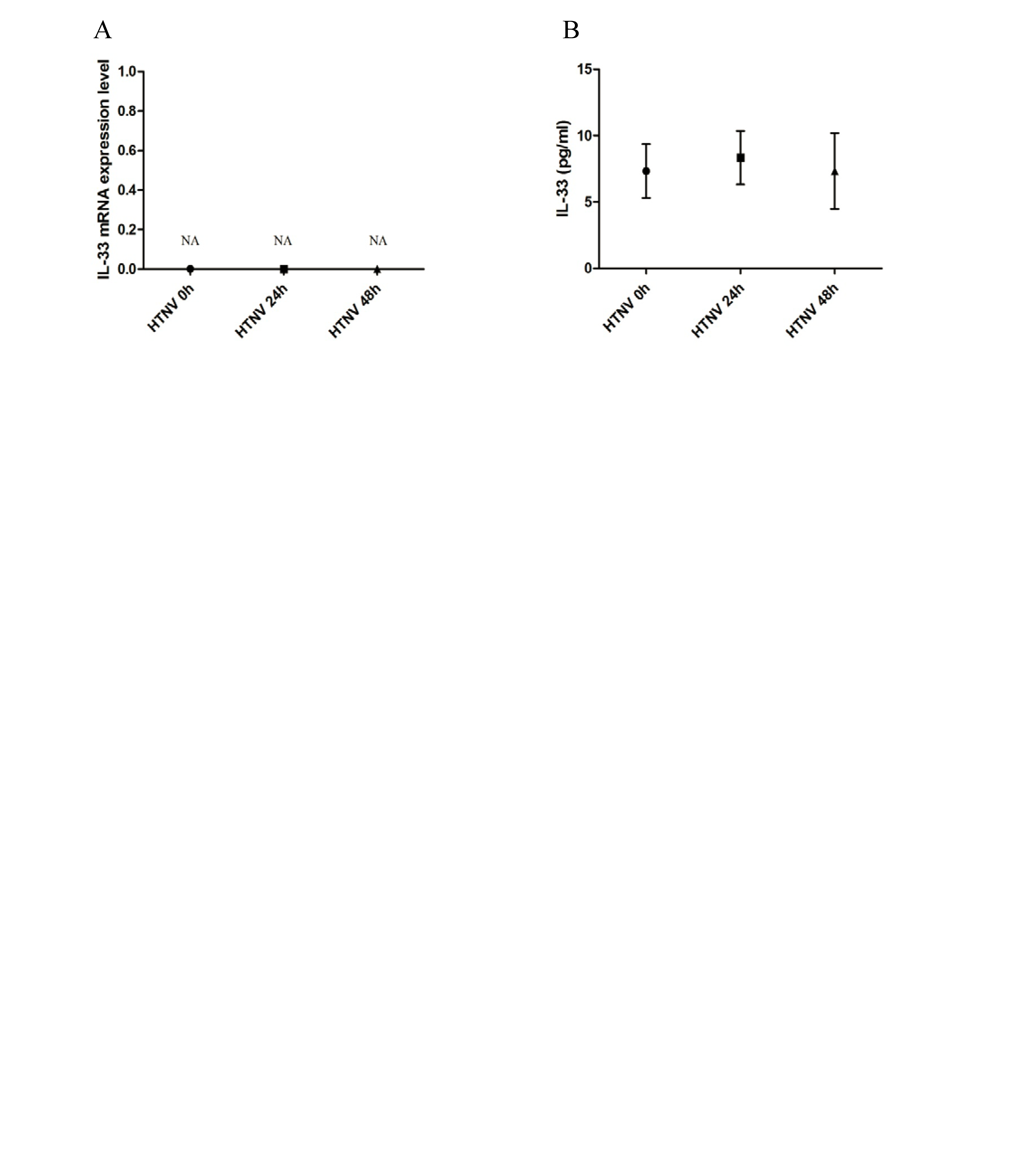

Supplement: S2 Fig — HUVECs were infected with HTNV (MOI = 1) for 0 h, 24 h, or 48 h, respectively. The mRNA level of IL-33 was determined by real-time PCR (A). The protein level of IL-33 was detected in the supernatant of the HUVECs by ELISA (B). NA: none of any. (TIF) [file pntd.0003514.s004.tif]

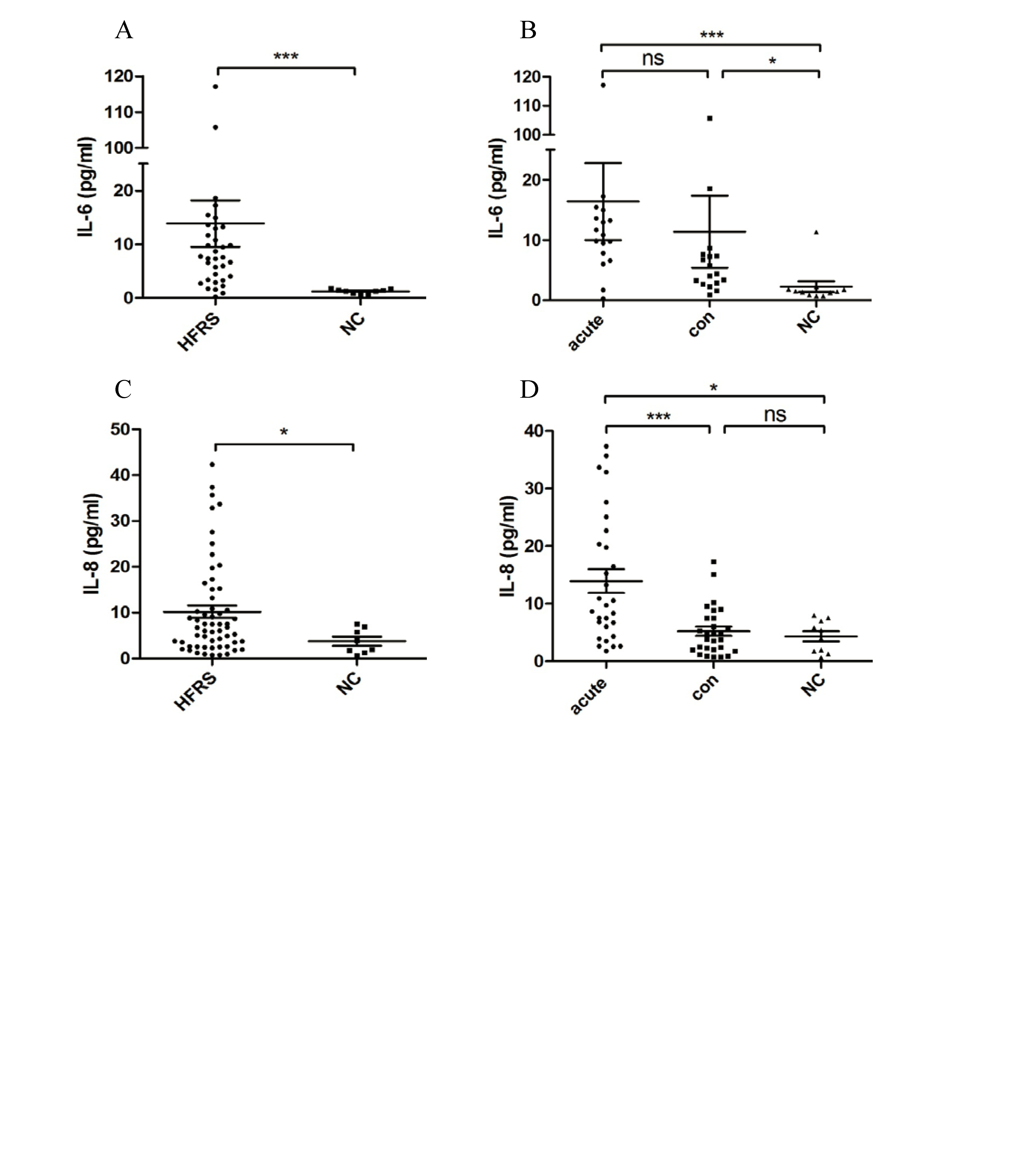

Supplement: S3 Fig — Scatter diagram displaying the protein levels of IL-6 and IL-8 in the plasma of HFRS patients detected by the ELISA kits (eBioscience, USA). Comparison of plasma IL-6 (A) and IL-8 (C) contents between HFRS patients and healthy donors (NC). Contents of IL-6 (B) or IL-8 (D) in the acute phase of HFRS (including febrile, hypotensive, or oliguric stage), the convalescent phase of HFRS (including diuretic or convalescent stage), and healthy donors (NC). Data are the means±SE (for IL-6, HFRS, n = 34; NC, n = 9; for IL-8, HFRS, n = 58; NC, n = 9), *p < 0.05, ***p < 0.001, HFRS patients versus NC or acute phase versus convalescent phase and NC. (TIF) [file pntd.0003514.s005.tif]
